# The Evolution of Telepractice Use during the COVID-19 Pandemic: Perspectives of Pediatric Speech-Language Pathologists

**DOI:** 10.3390/ijerph182212197

**Published:** 2021-11-20

**Authors:** Ying Hao, Saijun Zhang, Austin Conner, Na Youn Lee

**Affiliations:** 1 Department of Communication Sciences and Disorders, University of Mississippi, Oxford, MS 38677, USA; yinghao@olemiss.edu; 2 Department of Social Work, University of Mississippi, Oxford, MS 38677, USA; amconner@go.olemiss.edu (A.C.); nlee2@olemiss.edu (N.Y.L.)

**Keywords:** children, telepractice, service disruption and transition, COVID-19, Speech-Language services

## Abstract

The study investigated how pediatric speech-language pathologists (SLPs) applied telepractice to compensate for the loss of in-person services and the dynamics of telepractice use during the COVID-19 pandemic in a rural state. We conducted interviews with 10 SLPs and then a statewide survey in which 51 SLPs participated. The qualitative interviews revealed themes including changes in service environment due to the pandemic (e.g., transition to telepractice, losing clients), challenges in the transition to telepractice (e.g., limited training, difficulty engaging clients), worsening wellbeing of clinicians and clients, and SLPs’ perspectives and suggestions towards telepractice in the future. Survey results showed service disruptions and transition dynamics during the pandemic. SLPs’ weekly caseloads reduced from an average of 42.3 clients prior to the pandemic to 25.9 and 23.4 from March to May and from June to September 2020, respectively, and then recovered to 37.2 clients from October to December 2020. In contrast, the number of telepractice caseloads sharply increased from 0.2 clients per week prior to the pandemic to 14.8 from March to May 2020. The weekly telepractice caseloads then declined to 5.5 clients from June to September and 7.9 clients from October to December 2020. In the months right after the pandemic outbreak (i.e., March to May), client children struggled with treatment gains and behavioral wellbeing. However, their outcomes gradually improved by October to December and approached pre-pandemic levels. About one-third of the SLPs reported that they would be more likely or much more likely to use telepractice in the future regardless of the pandemic. However, only about a quarter perceived telepractice as comparable to in-person services. We concluded that the transition from in-person services to telepractice substantially mitigated service disruptions right after the pandemic outbreak and that telepractice’s substitute role evolved over time.

## 1. Introduction

The COVID-19 pandemic has seriously devastated the economy and altered people’s lifestyles [1]. The lockdown and other social distancing measures have resulted in extended isolation and loneliness, which can have profound impacts on people’s psychological and physical wellbeing (e.g., López-Bueno et al., 2020) [2,3]. While the public health crisis may have a pervasive negative influence on the population, children with Speech-Language disorders, who require extensive services to maximize their development, may be more severely affected (e.g., Chadd et al., 2021) [4]. Speech-Language pathologists’ (SLPs) perspectives are important for us to understand how the pandemic has been influencing service provision and wellbeing of children with Speech-Language disorders.

### 1.1. Service Disruption and Telepractice as an Alternative to In-Person Services

Prior to the pandemic outbreak, speech and language services were generally delivered in-person. A survey by the American Speech-Language–Hearing Association showed that more than 95% of SLPs solely utilized in-person services before the pandemic [5]. The pandemic outbreak interrupted in-person service delivery due to the need for extensive social distancing. As a result, some clients have encountered reduced services or even service loss. For example, Chadd et al.’s (2021) [4] national survey in April 2020 among SLPs in the United Kingdom showed that the number of referrals for Speech-Language services reduced by about one-third from the previous year.

The transition from in-person services to telepractice became prevalent from the onset of the pandemic. Telepractice incorporates a variety of telecommunication technologies, such as synchronous audiovisual technologies and asynchronous transmission of therapy materials via online platforms [6]. Prior to the pandemic outbreak, telepractice was especially appealing to clients residing in rural areas with scarce healthcare resources because of its unique advantage of overcoming geographical distances [7,8]. When social distancing restrictions made in-person speech and language services excessively difficult, telepractice came to the rescue. Sylvan et al., (2020) [9] surveyed 280 school-based SLPs across the United States in May 2020, finding that about three-quarters of the SLPs reported a transition to telepractice. Similarly, Campbell and Goldstein’s (2021) [10] survey in September 2020 showed that 87% of pediatric SLPs provided telepractice services in the months following the pandemic outbreak, compared to only 18% prior to the pandemic outbreak.

While a transition from in-person services to telepractice is apparent, details regarding how telepractice compensated for the reduction in in-person services are still unclear. This is especially true concerning the dynamics of using telepractice and in-person venues for speech and language service delivery along with the pandemic evolution, which limits a precise documentation and a deep understanding of SLPs’ experiences at this particular moment in time.

In addition, telepractice and in-person services may have a trade-off between convenience and service efficacy, which deserves an evaluation during the widespread transition from in-person services to telepractice. Weidner et al., (2020) [11] reviewed studies between 2014 and 2019 that examined the efficacy of telepractice-based Speech-Language services and found that telepractice was generally effective in screening, assessment, and treatment; however, most of these studies did not compare telepractice with in-person services. Among studies that made such comparisons, the findings generally suggest that telepractice and in-person services are comparable (e.g., Wales et al., 2017) [12]. However, skepticism remains. The large-scale transition provides an opportunity to study telepractice efficacy relative to in-person services.

The pandemic outbreak not only required SLPs to rearrange familial routines but forced them to make prompt transitions from in-person services to telepractice and/or restructure in-person service procedures in accordance with safety measures. Most SLPs did not have experience and training with telepractice and had to undergo a challenging trial-and-error learning process for the implementation of telepractice, which likely was further complicated by some client families’ lack of internet access and technology literacy. Moreover, client children’s characteristics, such as young age and/or substantial behavioral/emotional problems, may have posed difficulties in the implementation of telepractice. These may be further complicated by the need to manage in-person services for some clients under strict safety protocols. It is important to examine these challenges closely to advance our understanding of contexts for SLPs’ telepractice during the pandemic.

### 1.2. Client Family and Clinician Wellbeing during the Pandemic

The service disruption and transition have resulted in worsening wellbeing in children with disabilities. Nonweiler et al., (2020) [13] showed that a group of children with neurological disorders showed more severe emotional and behavioral problems (e.g., hyperactivity) after the pandemic outbreak than a group of children with similar characteristics before the pandemic. Even prior to the pandemic, research indicated that parents of children with disabilities were under excessive strain and had a higher risk of mental health problems compared to parents of typically developing children (e.g., Scherer et al., 2019) [14]. The pandemic may have further intensified these parents’ stress because of the challenges of accommodating children’s special needs in the difficult environment [15].

SLPs faced great challenges and stress when dealing with service disruptions and transitions. The abrupt changes may have forced them to promptly develop modified protocols for in-person and/or telepractice services [16]. Balancing work and family during this period may be an excessive challenge for SLPs [4]. Sylvan et al.’s (2020) [9] survey showed that about three-quarters of the SLPs reported an increased workload to adapt to the service transition due to the pandemic, with about one-quarter of them feeling that the increased workload was hard to manage. Most of the SLPs also expressed concerns about their own or family members’ health, and about one-quarter of them experienced financial stress.

### 1.3. The Rural Context of Service Disruption and Transition

The challenges of service disruptions and transitions can be especially pronounced for SLPs and their client children and families residing in rural, low-income areas, such as in the state of Mississippi. The state of Mississippi has a population of about three million residents and a population density of 63 people per square mile, which is 85% below the national average [17]. The state has the highest poverty rate in the nation, with nearly 20% of residents living below the poverty line [17], and has a shortage of healthcare resources [18]. According to Health Resources and Services Administration’s healthcare resources classification, 82% (67/82) of the counties in Mississippi are designated as rural counties, 2.4% (2/82) are partially rural, and only 15.9% (13/82) are non-rural [19]. While the state has limited healthcare resources, the rate of disabilities is high, with the Mississippi Delta region having the highest rate of disabilities in the nation [20]. The imbalance between resources and needs may be intensified during the pandemic, resulting in more severe service disruptions and challenges in service transformations. To the best of our knowledge, the impact of the pandemic on Speech-Language services has not been studied in a rural context.

### 1.4. Rapid Evolution of COVID-19-Related Policies

The COVID-19 pandemic has been evolving rapidly, and relevant state policies have been changing accordingly, which can have a direct influence on Speech-Language service practices. Taking the state of Mississippi as an example, the state declared a state of emergency in mid-March 2020 [21] and issued a statewide shelter-in-place order effective in early April 2020 until the end of the month [22,23]. In the same month, the state also decided to close schools for the academic year of 2019–2020 [24]. During the summer of 2020, school districts were asked to develop individualized plans to reopen for the 2020–2021 academic year, which may be in the form of in-person, virtual, and/or a combination of both [25]. Given the rapid evolution of the pandemic and responding policies, it is important to study the dynamics of telepractice and in-person venues for Speech-Language services.

### 1.5. The Current Research

While extant studies showed substantial disruptions and transitions in Speech-Language services during the COVID-19 pandemic, details remain unclear. To further the understanding, we examined the following questions in the current study: (a) How did telepractice compensate for the loss of in-person services? (b) What challenges had SLPs encountered during the service disruptions and transitions? (c) How did SLPs perceive telepractice as compared to traditional in-person services? (d) How were the service disruptions and transitions related to SLPs’ as well as client children and families’ wellbeing? Importantly, for the first time, we studied how service delivery venues have changed over time along with the rapid evolution of the pandemic in a rural context.

To achieve the study goals, we first conducted a primarily qualitative interview study in July 2020 with pediatric SLPs in Mississippi. On the basis of the interview study, we designed and conducted a quantitative survey in December 2020 targeting all SLPs in the state, in which SLPs were asked to retrospectively report service-related issues at several time points before and after the pandemic outbreak. In the following, we reported the interview study and the survey study, respectively. The research protocol was approved by the Institutional Review Board at the authors’ university.

## 2. Interview Study

### 2.1. Participants

A recruitment message was sent to SLPs practicing at four university clinics, an early intervention program, two private practices, four schools, and one hospital to maximize the representativeness of the sample in major SLP work settings [5]. The agencies in each setting category were randomly selected. Eligible participants were SLPs who provided services to children aged between 0 and 17 years with developmental disabilities, which include attention-deficit/hyperactivity disorder (ADHD), autism spectrum disorder, intellectual disability, hearing loss, vision impairment, learning disability, and other developmental disabilities (children with developmental disabilities is an umbrella term referring to the variety of pediatric populations that SLPs serve in different settings; in the recruitment message, we made it clear to the SLPs that they were expected to report service experiences and perspectives related to their client children and families) [26]. A total of 11 SLPs responded to the invitation. Upon receiving a response from an SLP, an interview was scheduled, and a trained PhD student subsequently conducted the interview. Ten SLPs completed the interview and one SLP did not respond to follow-up requests after an initial response. A USD 30 Amazon e-gift card was delivered to each participant soon after the interview. See Table 1 left panel for the demographic information of the interview sample.

The interviews were conducted via Zoom or telephone and were audio recorded. If the interviews were conducted via Zoom, the interviewer disabled the video function and only recorded the audio of the interview. The interviewer used name initials to refer to the participants during the interview to enhance identity protection.

### 2.2. Instrument

We designed a two-section data collection instrument for the interview, including a close-ended question section and an open-ended question section. In the closed-ended question section, the interviewer collected participants’ demographic information, quantitative estimates of total and telepractice client numbers in a typical week before and after the pandemic outbreak, telepractice training, and confidence levels with telepractice. In the open-ended question section, the interviewer asked a series of preset questions to elicit more in-depth and extensive information on participants’ personal experiences with service disruptions, transitions to telepractice, and the wellbeing of clients and themselves during the pandemic. The interviews were generally completed within 40 min.

### 2.3. Analytical Strategies

We used thematic analysis to identify themes across interviewees’ responses [27]. The first author and two research assistants (RAs) listened to all the recordings to immerse in the data. Each RA then transcribed half of the interviews, and a third RA listened to all the recordings and checked the transcripts to ensure accuracy. Next, the first author and the two RAs read all the transcripts repeatedly and produced initial codes. The three met several times virtually to review the initial codes based on the transcripts. On the basis of the discussion, they determined a set of updated codes by collapsing similar codes (e.g., “clinician lacked training of telepractice” and “client parent lacked training of telepractice” were combined into “clinician/client lacked training of telepractice”) and reached consensus on the codes. The three then applied the codes to the transcripts independently and met virtually to address coding discrepancies for the entire dataset. The third RA was asked to match 20 randomly selected quotes and corresponding codes, which yielded a full matching. On the basis of the finalized codes and quotes collated together within different codes, the three RAs then further met to identify key themes and subthemes through asking questions [28]. For example, what is the relationship of one code to another? Can they be grouped together for an upper-level category? The themes and subthemes were reviewed and defined to be sufficiently abstract to represent the codes and quotes.

### 2.4. Results

Each clinician, on average, experienced a decline in their weekly total caseloads by about one-third, from 34.7 (SD: 17.3) to 23 (SD: 12.8) clients four months into the pandemic. Telepractice weekly caseloads, on the other hand, increased on average from 0.5 (SD:1.3) before the pandemic outbreak to 16.6 (SD: 12.2) clients after the pandemic occurred. Before the pandemic, only one clinician used telepractice frequently, and the remaining had no use or little use of telepractice. However, at the time of the interview, eight clinicians used telepractice quite frequently or almost all the time, and two used a little. Although half of the clinicians did not receive any formal training (e.g., a course systematically introducing telepractice) prior to the pandemic outbreak, confidence levels were high (on average 4.7 on a five-point scale rating ranging from very unconfident (1) to very confident (5)) at the time of the interview. Regarding responses to the open-ended questions, five key themes were identified, and subthemes were identified for four of the five key themes. Details about the codes related to the themes and subthemes are presented in Appendix A.

#### 2.4.1. Theme 1: Changes Due to the Pandemic

Major changes due to the outbreak of the pandemic included transitioning to telepractice, losing clients, child client regression on previously acquired skills, increased emotional and behavioral problems, a gap in service after the pandemic occurred, and a shorter duration for in-person sessions.

One clinician reported that telepractice increased demands on parents, which contributed to client loss: “If parents are having to be too involved and it is too overwhelming for them, we have had loss of clients that way or at least some temporary disruptions in service until we could figure out another way”.

Clinicians also observed increased emotional and behavioral problems among client children: “I had kids that just lost the structure and lost the routine and even though they are very young, you would think that they wouldn’t be that in tune but they were…… We saw a lot of increased behavioral aspects and emotional aspects”.

Other changes were less commonly reported but they include client routine disruption, disinfection needs for clinicians providing in-person services (e.g., “you spend a lot of time disinfecting everything, disinfecting the room and yourself”), and a different way of presenting materials and activities (e.g., “pull in things like pictures that will pop up on the tablet and music videos”).

#### 2.4.2. Theme 2: Telepractice Is Challenging

Clinicians shared their experiences of using telepractice to deliver Speech-Language services, and it appeared that more challenges of telepractice were reported than its benefits. Almost all clinicians reported limited training on telepractice for themselves and/or the client parents. The scope of training goes beyond technology to the abilities of offering intervention via telecommunication and training parents as service facilitators.

Some clinicians had a low acceptance of telepractice due to their lack of confidence in providing telepractice services and doubt in its efficacy. One clinician shared, “I did not feel confident in my ability to provide the services, and I didn’t feel confident in directing my students on exactly how to do it”. Clinicians also reported that some parents did not have confidence in telepractice services. “Therapist thought yeah sure this [telepractice] could work well, it could be a great thing for the patient, the parents didn’t think their child would take to it”.

Compared to in-person sessions, telepractice sessions were regarded as less hands-on and did not easily engage children, especially younger clients with relatively short attention spans. One clinician gave an example, “I started using a green screen because Zoom lets you change the background, but for the younger kids I would have to get very theatrical and super silly and dramatic and find ways to make the toys and pictures, kind of, almost like more visually stimulating”. In addition, limited internet access, device availability, client family readiness, more demands on parents, and clinician adjustment to design and plan telepractice sessions were common challenges.

Other barriers were relatively less commonly mentioned, including limited insurance coverage for telepractice, rapport building with new clients, and that “it was easy for the parents to forget” teletherapy appointments. Despite the challenges, some SLPs reported benefits of switching to telepractice, such as improved service outcomes for some children, increased parent involvement, and more convenience.

#### 2.4.3. Theme 3: Worsening Wellbeing of Clinicians and Clients

SLPs reported deteriorated wellbeing among both themselves and clients. Seven clinicians reported increased stress, but five of them reported that stress levels were minimized once they were accustomed to the situation. “At the very beginning it was very stressful because the unknown is I think the hardest thing…… Now I think we are in a stage where it is less of a reaction of having to deal with a problem to more of [an] okay how do we transition and more forward and live with this”.

Clinicians also observed stressful parents during the difficult time period. “I think people are getting sick and you’re seeing the COVID spread that parents are so concerned for their child that…I’ve experienced some situations where I feel like a parent is taking their stress and anxiety out on me because it feels like we’re the only place these parents go or interact with someone or anything that has to do with their kids”.

#### 2.4.4. Theme 4: Telepractice Should Continue When Appropriate

Despite the challenges of telepractice, nine out of the ten clinicians hoped that telepractice would continue to be an option for future Speech-Language services. However, they noted various issues, such as insurance coverage and child client engagement, that need to be addressed to advance telepractice development. Moreover, they perceived telepractice to increase access to Speech-Language services for underserved populations, such as people residing in rural communities. “I am hoping this [telepractice] is going to show their value, and, especially reaching out to underserved populations. You know, to rural communities that don’t have access. So…what is going to come is that the benefit of telepractice for a lot of different situations is going to be seen and it will continue and we’ll have to find ways to make sure it’s funded and paid for by insurance companies”.

Only one clinician expressed negative feelings towards telepractice due to concerns about efficacy, child engagement, and shallow learning related to telepractice. “After my experiences with it and the decreased efficacy of my applied therapy, it just doesn’t feel the same in terms of engaging a client in order to, essentially teach them, or engage them to help them develop their skill. Especially with modern technology now, in kids engaging with screens, I feel like the depth of engagement and the processing and perception of what they’re learning from a screen is shallow and not by any fault of the child or the parent or myself”.

#### 2.4.5. Theme 5: Suggestions for Future Services

Clinicians made many suggestions for future telepractice and in-person services. For future telepractice, the most common suggestion was that technology support should be provided to families, such as internet connection, devices, and parent technology training. One clinician expressed: “I think all of our families have to have access to good internet, that has been a problem for several kids that I see. I have one family that gets in the car and drives to sit outside at a place so the child has to have therapy in their car to have access”.

Other suggestions by a few clinicians were offering a hybrid model with both teletherapy and in-person therapy and increasing insurance coverage for telepractice. “We need all of our insurances on board to understand that teletherapy is not subpar practice by any means and that [it] can be very beneficial”. There was also an expressed need to strengthen research for evidence-based practice in telepractice. In addition, there were a few recommendations for future in-person services, mainly about scheduling adjustments due to the increased cleaning time.

## 3. Survey Study

### 3.1. Participants

On the basis of the findings from the interview, we designed a quantitative survey to target SLPs practicing statewide in Mississippi in December 2020. We first emailed a recruitment message to all the 803 certified SLPs in the state who opted to receive messages via the American Speech-Language–Hearing Association member directory. The invitation specified that we were recruiting SLPs who served children with developmental disabilities aged 0 to 17 years in the state of Mississippi. The invitation message resulted in responses from 57 SLPs who were willing to participate in the survey, which accounted for a 7.1% response rate. We then delivered a unique, anonymous survey link through the Qualtrics survey system to each of the prospective participants and sent two reminders in the following weeks. At the end of December 2020, 51 of the SLPs completed the survey. A USD 10 Amazon e-gift card was sent to each of them. See Table 1 the right panel for the demographic information of the survey sample.

### 3.2. Instruments

An anonymous online survey was administered with questions similar to the closed-ended questions from the interview. We specified four time-phases (i.e., pre-pandemic, March to May 2020, June to September 2020, October to December 2020) for questions about service delivery status so that potential changes over time could be estimated. Additionally, on the basis of SLPs’ interview responses, we asked questions about speech-language treatment gains, children’s behavioral and emotional problems, caregiver engagement, and clinician and client stress levels throughout the three periods of the pandemic (i.e., March to May 2020, June to September 2020, October to December 2020). IBM SPSS Statistics 25 was used for quantitative analyses.

### 3.3. Results

Compared to the number of 42.3 prior to the pandemic, SLP’s weekly caseloads declined to 25.9 between March and May 2020 and 23.4 between June and September 2020, but bounced back to 37.2 between October and December 2020. Repeated measures ANOVA showed a main effect of time (F (2, 116) = 20.71, *p* < 0.01). Pairwise comparisons using Bonferroni corrections indicated that total caseloads of different periods were different from each other (*p* < 0.05), except for two pairs of periods, pre-pandemic and October–December (*p* = 0.07), and March–May and June–September (*p* = 1.0).

**Figure 1:**
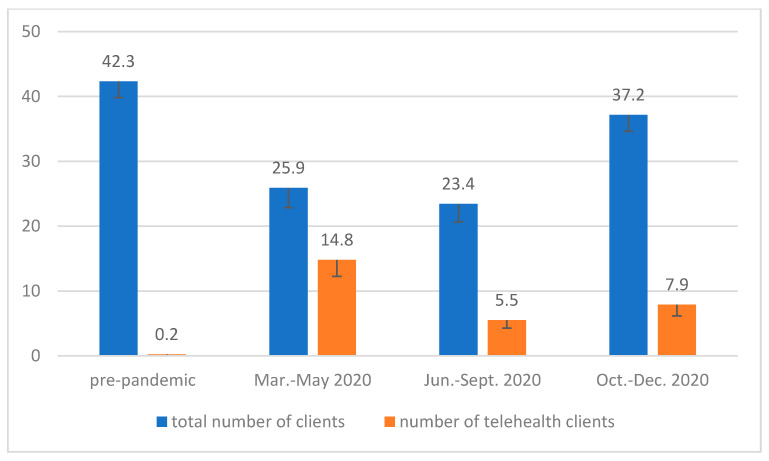
Total number of clients and number of telepractice clients in a typical week before the pandemic and during the pandemic from the December 2020 survey (*n* = 51).

Over the course of the pandemic, the weekly telepractice caseload increased from 0.2 to 14.8, dropping to 5.5 and 7.9 in the following periods. The main effect of time was significant (F (2, 81) = 12.11, *p* < 0.01). Pairwise comparisons using Bonferroni corrections indicated that each pair of periods was different at *p* < 0.05, except for two pairs, March–May and October–December (*p* = 0.23), and June–September and October–December (*p* = 1.0) (see Figure 1).

**Figure 2:**
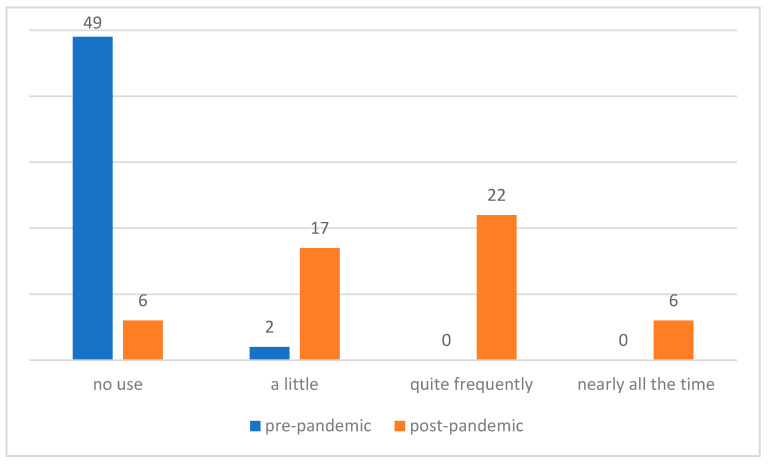
Number of SLPs who used telepractice before and during the COVID-19 pandemic in the December 2020 survey (*n* = 51).

Before the pandemic, only two (3.9%) SLPs had little use of telepractice. At the time of the survey, 45 (88.2%) of the SLPs used telepractice, and 28 (54.9%) used it quite frequently or nearly all the time. The difference before and after the pandemic outbreak is significant (χ^2^ (3, 51) = 73.46, *p* < 0.01) (see Figure 2).

Regarding training on telepractice, 35 (68.6%) clinicians did not receive formal training before and during the pandemic. During the three periods from March to May, June to September, and October to December in 2020, SLPs’ rating of their confidence in telepractice on a five-point scale (from very unconfident (1) to very confident (5)) increased from 1.93 (SD: 0.89) to 3.0 (SD: 0.92) and 3.7 (SD: 0.96), respectively. A main effect of time was found for SLPs’ confidence (F (2, 60) = 74.8, *p* < 0.01), and each pair of periods was significantly different (*p* < 0.05).

Regarding overall perceptions towards telepractice, 15 (29.4%) SLPs were more likely or much more likely to use telepractice in the future, and 13 (25.5%) viewed the two service delivery venues as comparable. While over two-thirds of the clinicians did not think that telepractice was comparable to in-person services, about half of these clinicians agreed that telepractice was a good option for some purposes (e.g., consultation, basic care) (see Table 2).

**Figure 3:**
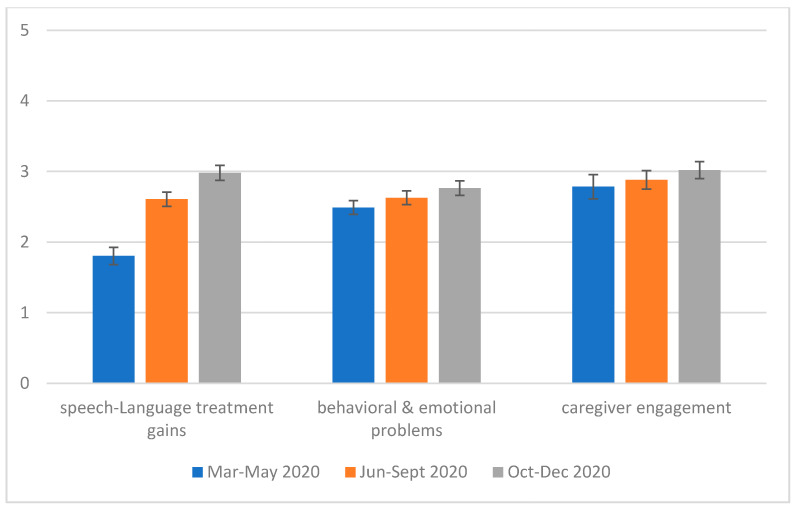
Longitudinal changes for Speech-Language treatment gains, behavioral and emotional problems, and caregiver engagement across the three periods during the pandemic in the December 2020 survey (*n* = 51). *Notes:* SLPs rated the three aspects on a five-point scale: 1 = much less/worse than pre-pandemic level, 2 = less/worse than pre-pandemic level, 3 = similar to pre-pandemic level, 4 = more/better than pre-pandemic level, and 5 = much more/better than pre-pandemic level.

Compared to the pre-pandemic period, service treatment gains for the child clients were the smallest between March and May 2020. The gains gradually bounced back, and during the period of October–December 2020, it reached a level similar to that of the pre-pandemic period. A similar trend was found in the improvement of children’s behavioral/emotional problems and of caregivers’ level of engagement in the interventions. There were main effects of time for Speech-Language treatment gains (F (2, 86) = 77.5, *p* < 0.01; each pair was different at *p* < 0.05) and behavioral and emotional problems (F (2, 100) = 3.51, *p* = 0.03; yet none of the pairs was significantly different at *p* < 0.05 after the Bonferroni corrections). For caregiver engagement, the main effect of time was not significant (F (2, 76) = 1.8, *p* = 0.17) (see Figure 3).

Finally, the stress levels of SLPs, which were rated on a four-point scale (from none (1) to severe (4)), declined from 2.96 in March–May to 2.42 in June–September, and remained stable at 2.42 in October–December. As rated by clinicians, caregivers’ stress levels also declined, from 3.82 in March–May, to 3.12 in June–September, and further to 2.98 in October–December on a five-point scale (from not stressed at all (1) to extremely stressed (5)). There were main effects of time for both SLP stress (F (2, 98) = 10.60, *p* < 0.01) and caregiver stress (F (2, 64) = 10.22, *p* < 0.01), and for both measures, each pair of periods was different at *p* < 0.05, except for the pair of June–September and October–December (*p* = 1.0).

## 4. Discussion

This study explored the impact of the COVID-19 pandemic on pediatric Speech-Language services. Results from an interview study in July 2020 and a survey study in December 2020 among pediatric SLPs revealed details in service disruptions and telepractice compensations for in-person services. Importantly, this is one of the first studies that have examined the dynamics of service disruptions and recovery amid the rapid evolution of the pandemic in a rural context.

### 4.1. Service Disruption and Transition

Consistent with previous studies [4,9], current findings indicate substantial service disruptions due to the pandemic. In the early stage of the pandemic, clinicians reported a client reduction of about 30% to 40% in both the interviews and surveys, indicating that the magnitude of service disruptions for children with Speech-Language disorders was striking during the first several months after the pandemic outbreak in 2020.

While it is typical for an unexpected global pandemic to strain healthcare and other services, the impact is likely more pronounced in rural areas due to a serious shortage of healthcare resources. Moreover, a lack of access to the internet and devices, loss of employer-sponsored health insurance coverage [15], and cumbersome procedures to obtain approval for teletherapy from insurance companies, as noted by some of the interviewed clinicians, appear to have substantially disrupted services.

From the interview, most clinicians viewed a lack of internet access or poor internet connection as a serious barrier to telepractice. Despite the rapid infrastructure development, in 2019, 20% of residents in the state of Mississippi did not have access to standard broadband, relative to only 7% nationwide [29]. Although accelerated infrastructure development can increase internet availability in Mississippi in the near future [30], the cost of broadband services, which may account for more than 5% of household income for low-income families [31], may still restrict its accessibility. In addition to increased funding for broadband infrastructure development, policymakers may need to consider subsidizing low-income families’ internet service use, which is critical to the dissemination of telepractice to underserved populations.

The findings also shed light on the transition from in-person services to telepractice after the pandemic outbreak. There was only one clinician among the 10 interviewed used telepractice frequently before the pandemic. The findings remained consistent in the statewide follow-up survey, with only two of the 51 clinicians having used telepractice before the pandemic. This reflects a common situation: Speech-Language services and other behavioral services were primarily offered in person before the pandemic [32]. Since the pandemic happened, all interviewed clinicians and nearly all the surveyed clinicians (96%) were using telepractice. However, as indicated in the survey, while more than half of the clinicians used telepractice frequently or nearly all the time, more than two-fifths reported infrequent use or no use after the pandemic outbreak. This suggests that there was substantial variation in the degree of telepractice utilization among SLPs during the pandemic, and future research can further explore the underlying reasons.

Half of the interviewed clinicians reported that they did not receive formal training for telepractice. The percentage for the surveyed clinicians was even higher (69%). This is consistent with the findings from previous survey studies (e.g., Sylvan et al., 2020) [9]. Among the interviewed clinicians, many said they were not confident with telepractice at the beginning of the pandemic, but all felt quite confident at the time of the interview several months into the pandemic. This was further confirmed in the follow-up survey. We observed a low level of confidence in telepractice during the period from March to May 2020, but it continued to increase throughout the following two periods and reached a generally confident level by the end of 2020. The clinicians in both samples had around 14 years of experiences in Speech-Language services on average, which may help facilitate their transition to telepractice. Although trial and error help with the learning of telepractice, most SLPs considered that formal training for telepractice is important [33]. Skills such as nonverbal communication, information delivery, and rapport building are different in telepractice and in-person services. Thus, enhanced telepractice training is necessary in acquiring these skills [34,35].

### 4.2. Strengths and Weaknesses of Telepractice

The interviewed clinicians offered valuable insights into the strengths and weaknesses of telepractice. Clinicians reported that compared with in-person services, telepractice was more challenging in engaging children in activities. In telepractice, the need of presenting materials and activities via a screen is quite different from traditional in-person services. Some facilitating strategies are important for telepractice, such as the assistance of e-helpers to build rapport and engage children. The clinicians also pointed out that parents were more likely to face increased assistance demands. It may be necessary for the clinicians to help parents prepare for telepractice in advance, such as helping them prepare for a list of materials for therapeutic sessions and suggesting them to select an appropriate room to minimize distractions to improve service efficiency.

The clinicians noted some unique strengths of telepractice for some, if not all, client families. Convenience was a big advantage of telepractice, such as saving traveling time and serving rural families easily. Some parents appeared to be more involved in telepractice than in in-person services, and some parents had more interactions with their children after working from home.

### 4.3. Perceptions of Telepractice

When examining clinicians’ perspectives toward telepractice use in the future when the pandemic fades away, we found some notable differences between the interviewed clinicians and surveyed clinicians. While the interviewed clinicians were generally optimistic, half of the surveyed clinicians reported that they were unlikely or very unlikely to use telepractice in the future when the pandemic ends. This may be related to the sample difference between the interview and the survey study. A large percentage of clinicians who were interviewed worked in community agencies, mainly university clinics (40%), whereas most clinicians who completed the survey worked in schools (57%). It is possible that the SLPs working in university clinics were more open to telepractice than SLPs in other settings, given that they tend to have more exposure to research and pilot studies applying telepractice. Another factor that may have contributed to the perception discrepancy is timing. The interview was conducted in July 2020 when the nation was under a serious pandemic threat and tight social distancing restrictions, resulting in the SLPs’ highly positive view toward telepractice. By December 2020, when the survey was conducted, people were more used to the pandemic environment; the promise of vaccines and loosened restrictions would have eased people’s concerns and facilitated in-person services [36]. Therefore, SLPs may have shifted their perceptions and did not think that they need to heavily rely on telepractice in the future.

Nonetheless, about one-third of the surveyed clinicians said they were more likely to use telepractice post-pandemic than pre-pandemic. SLPs’ experiences with telepractice have allowed them to learn telepractice’s benefits, gain telepractice skills, and increase their confidence in telepractice, which may have led them to be more favorable toward telepractice in the future. Therefore, we speculate that clinicians are more inclined to integrate telepractice in service delivery in the future. However, since the use of telepractice is still undergoing a dynamic process as the pandemic and societal responses evolve, future research should continue investigating its roles during and beyond the pandemic environment.

### 4.4. Clinician and Client Wellbeing

The pandemic negatively affected the wellbeing of clinicians. In addition to facing the universal pandemic threat and stress, most of the interviewed clinicians were under pressure to make swift changes. Moreover, continuing in-person services may have exposed them to great risks for infection. Fortunately, some interviewed clinicians reported that their stress declined when they got used to the new routine. The survey findings were consistent with the interview results. Clinicians’ stress levels were higher in the first three months right after the pandemic outbreak, but they substantially reduced and remained generally stable in the following periods. However, in view of the lasting pandemic, the accumulated stress may still impose serious harm on the wellbeing of clinicians, and therefore service agencies should consider timely support and resources to mitigate such stress [37].

The pandemic also has a significant impact on the wellbeing of client children and their families. Children are more vulnerable to environmental stress, such as the pandemic threat, due to their developmental stage. Isolation and loneliness due to quarantine and social distancing during the pandemic can seriously affect their psychological and behavioral wellbeing [2,3]. López-Bueno et al., (2020) [2] extensively reviewed studies examining health-related behaviors for pre-school and school-age children subject to isolation to understand potential consequences due to COVID-19 lockdown. The findings show that extended isolation is often closely associated with adverse health behaviors, such as increased screen time, consumption of unhealthy foods, worsened mental health, reduced physical activities, and increased sedentary behaviors. Even though active online activities such as social media engagement may compensate for part of the lost social connections, the pandemic increased children’s anxiety and negative emotions as reflected in their social media expressions [36]. The impact on families of children with developmental disabilities can be intensified because of their service disruptions and transitions to telepractice, as well as the lack of adequate telepractice training and other assistance during the pandemic.

Moreover, more than half of the interviewed clinicians reported that client children regressed on previously acquired skills or developed more emotional and behavioral problems at home, possibly due to the service loss or gap during the transition from in-person services to telepractice. The follow-up survey offered more insights into these issues. Clinicians reported that in the first three months after the pandemic outbreak, caregivers of client children experienced more stress, but it gradually declined in the following months. Similarly, child behavioral and emotional problems were worst in the first three months after the pandemic outbreak, but there was improvement in the following two periods.

The findings corroborated the notion that health degradation among children with developmental disabilities and their parents is especially concerning when services are disrupted [15,37]. Substantial treatment gains at the end of year 2020 may be related to the improved competence with telepractice and the recovery of in-person services in the following months. It is possible, however, that the impact of service disruptions and transitions on children’s wellbeing is intermixed with that of isolation, loneliness, and other pandemic consequences. Future research can further detangle such impact by accounting for these potential confound factors. Regardless of the influential factors, mental health support is needed to help prevent client children and families from health deterioration.

### 4.5. Limitations

Limitations should be noted. First, the assessment of client wellbeing was reported by SLPs on the basis of their observations and interactions with the client families. Despite such secondary information, the findings are generally consistent with findings from studies that directly examined similar client populations (e.g., Patrick et al., 2020) [15]. It is worthwhile for future research to collect data directly from children and their families who receive speech and language services to verify and extend current findings in this aspect.

Second, we did not collect information on whether the SLPs provided services to rural or urban residents, limiting us from assessing how the service disruption and transition may disproportionally affect rural children and families. As the state’s primary care resources were mostly defined as rural, the findings should be more reflective of the service status in rural areas during the pandemic. Third, we used SLPs’ retrospective reflection to assess service changes across several phases before and after the pandemic outbreak, which may affect information precision. However, research shows that the recollection of important events years ago is still reliable, and therefore recall accuracy may not be a serious concern in this study given the relatively short recall period [38].

Finally, the study sample was from a rural state in the United States, and the sample sizes for the qualitative and quantitative studies are relatively small. Therefore, caution is needed when generalizing the findings to other regions. However, findings from the qualitative study offer triangulation for most findings in the quantitative study. The largely comparable findings from both the qualitative and quantitative studies and the consistency between findings from the current research and previous research suggest that the findings should be generally reliable.

## 5. Conclusions

We conducted an interview study in July 2020 and then a statewide survey study in December 2020 with pediatric SLPs in a rural state of the United States to investigate their experiences during the COVID-19 pandemic. The interview study showed that clinicians experienced a reduced total caseload but an increase in telepractice caseload. The clinicians reported a quick transition to telepractice, challenges and benefits of telepractice, worsening wellbeing of client children and their caregivers, an overall positive attitude towards telepractice, and suggestions for future telepractice and in-person services. The survey at the end of 2020 revealed the dynamics of service provision along with the pandemic evolution. SLPs experienced the most prominent service disruptions and transitions in the first two months after the pandemic outbreak. In the following months, however, their caseloads and intervention outcomes recovered steadily and approached pre-pandemic levels by the end of 2020. Most of the SLPs had no experience or training in telepractice prior to the pandemic outbreak and lacked confidence in applying telepractice at the beginning, but they eventually achieved a high level of confidence in the latter months of 2020. Although SLPs experienced some stress initially, their stress levels declined substantially a few months into the pandemic. Overall, the findings highlight the important and dynamic role of telepractice in substituting in-person services during the pandemic and the need to improve telepractice services. Meanwhile, it is also important to consider mental health support to client children and families, as well as clinicians, who encountered substantial stress in the pandemic environment.

## Figures and Tables

**Table 1:** Demographic information for the interview sample in July 2020 and the survey sample in December 2020.

Characteristics	Interview Sample (*n* = 10)			Survey Sample (*n* = 51)			*p* ^b^
	Mean/%	SD	Range	Mean/%	SD	Range	
Age (year)	37.7	8.2	26–51	38.8	11.9	25–67	0.77
Gender (female)	100%			100%			
Race							0.80
White	90%			88.2%			
African American	10%			7.8%			
Other	0%			4.0%			
Work setting ^a^							0.19
Community agency	40%			15.7%			
Private practice	30%			19.6%			
School	20%			56.9%			
Hospital (including outpatient)	10%			3.9%			
Multiple settings	0%			3.9%			
Years of working as SLP	14.4	8.7	3–30	13.8	11.6	1–45	0.89

Notes: ^a^ Community agency included university clinics and an early intervention program. ^b^ For the group comparison between interview participants and survey participants, we used *t*-tests for numeric variables and chi-squared tests for categorical variables.

**Table 2:** Clinician perception to telepractice as reported in the December 2020 survey (*n* = 51).

Measures	Percentage	Counts of SLPs
Likelihood of using telepractice in the future		
Very unlikely	17.6%	9
Unlikely	33.3%	17
Similar	19.6%	10
More likely	19.6%	10
Much more likely	9.8%	5
Do you feel that clients get comparable services through telepractice as they do for in-person services?		
Yes, comparable	25.5%	13
No, telepractice will never match in-person services	35.3%	18
No, but telepractice is a good option for some purposes	37.3%	19
Not sure	1.9%	1

## Data Availability

The study did not report any data.

## Appendix A

**Table A1:** Themes, subthemes, codes, and number of SLPs included each code when responding to the interview open-ended questions.

Themes	Subthemes	Codes	Number of SLPs
Changes due to the pandemic		Transition to telehealth	8
		Losing clients	6
		Client regression on previous acquired skills	6
		Client increased emotional and behavioral problems	5
		Service gap after the pandemic outbreak	5
		Shorter duration for sessions	4
		Client routine disruption	2
		Disinfection needs for clinicians providing in-person services	2
		Transition to a coach-parent model	1
		A different way to demonstrate intervention materials	1
Overall telehealth is challenging	Challenges	Less hands-on compared to in-person sessions	8
		Hard to engage some children	8
		Clinician/Parent lacked training of telehealth	8
		Clinician/Parent low acceptance of telehealth (e.g., lacked confidence, telehealth efficacy)	8
		Client family readiness	7
		Internet access	7
		Device availability	6
		Clinician adjustment to design and plan telehealth sessions	6
		More demands on parents	6
		Swift changes	5
		Limited insurance coverage for telehealth	4
		Hard to engage new clients without establishing rapport in person	3
		Hard to implement group sessions to facilitate peer interaction	3
		Hard to get quality evaluation results/standardized tests	3
		Hard to disseminate to rural households	2
		No physical proximity	2
		Easy for clients to forget appointment	2
		Increased preparation time for telehealth sessions	2
		Decreased interdisciplinary support (e.g., OT)	1
		Less effective supervision of graduate students due to that supervisor is in a learning process	1
		Harder to use AAC devices in teletherapy	1
		Too much screen time does not promote in-depth learning	1
	Benefits	Improved performance for some children through teletherapy	9
		More parent involvement to assist intervention	8
		More convenience (e.g., reaching rural families, no traveling)	8
		Parents could observe intervention strategies	6
		Able to serve more clients	5
		Having a window to understand how the families manage their situations at home	3
		Allowing for continued services without a big gap after the pandemic	3
		Addressing families’ needs at the moment	2
		More efficient real-time supervision for graduate students (e.g., using chat box to provide instant feedback at the moment)	1
Worsening wellbeing of clinicians and clients	SLP wellbeing	Increased stress levels	7
		Mitigated stress when getting used to the situation	5
		Balance between family and work	5
		Concerns about client wellbeing (e.g., adequate care)	5
		SLPs’ family burdens to take care of their own kids and spouses	4
		Potential financial burdens due to caseload reduction	3
		Home life being minimally affected	1
		Feeling unsafe getting back to in-person services	1
	Client wellbeing	Stressful parents during the difficult times	4
		Significant impacts on client social-emotional wellbeing	2
		Financial stress	1
Telehealth should continue in appropriate context	Optimism	Telehealth will continue in the future in appropriate context	9
	Pessimism	Telehealth will not continue due to concerns with its efficacy	1
Suggestions for future services	For telehealth services	Better internet connection for some families	6
		Continued telehealth training and more resources available for clinicians	3
		A hybrid model with tele- and in-person services to service clients	2
		Increased insurance coverage for telehealth that is comparable to in-person services	2
		Providing parent technology training	2
		Devices available for clients	2
		More research for evidence-based practice for telehealth	2
		Equity of services for clients with and without access to telehealth services	1
	For in-person services	Scheduling adjustment by taking into account of disinfecting time	3
		More supplies for disinfecting	1
		Clients adhering more to CDC guidelines	1
		Considering parents’ level of comfort	1
		Clinician controlling sanitation in clinic to ensure the safety of in-person services	1
